# High dietary quality of non-toxic cyanobacteria for a benthic grazer and its implications for the control of cyanobacterial biofilms

**DOI:** 10.1186/s12898-017-0130-3

**Published:** 2017-05-18

**Authors:** Sophie Groendahl, Patrick Fink

**Affiliations:** 1 Cologne Biocenter, Workgroup Aquatic Chemical Ecology, University of Cologne, Zuelpicher Strasse 47b, 50674 Koeln, Germany; 2 Institute for Zoomorphology and Cell Biology, Heinrich-Heine University of Duesseldorf, Universitaetsstrasse 1, 40225 Duesseldorf, Germany

**Keywords:** Herbivore, Balanced diet hypothesis, Nutrients, Nitrogen, Phosphorus, Compensatory feeding, Benthic algae, *Lymnaea stagnalis*, Fatty acids

## Abstract

**Background:**

Mass occurrences of cyanobacteria frequently cause detrimental effects to the functioning of aquatic ecosystems. Consequently, attempts haven been made to control cyanobacterial blooms through naturally co-occurring herbivores. Control of cyanobacteria through herbivores often appears to be constrained by their low dietary quality, rather than by the possession of toxins, as also non-toxic cyanobacteria are hardly consumed by many herbivores. It was thus hypothesized that the consumption of non-toxic cyanobacteria may be improved when complemented with other high quality prey. We conducted a laboratory experiment in which we fed the herbivorous freshwater gastropod *Lymnaea stagnalis* single non-toxic cyanobacterial and unialgal diets or a mixed diet to test if diet-mixing may enable these herbivores to control non-toxic cyanobacterial mass abundances.

**Results:**

The treatments where *L. stagnalis* were fed non-toxic cyanobacteria and a mixed diet provided a significantly higher shell and soft-body growth rate than the average of all single algal, but not the non-toxic cyanobacterial diets. However, the increase in growth provided by the non-toxic cyanobacteria diets could not be related to typical determinants of dietary quality such as toxicity, nutrient stoichiometry or essential fatty acid content.

**Conclusions:**

These results strongly contradict previous research which describes non-toxic cyanobacteria as a low quality food resource for freshwater herbivores in general. Our findings thus have strong implications to gastropod-cyanobacteria relationships and suggest that freshwater gastropods may be able to control mass occurrences of benthic non-toxic cyanobacteria, frequently observed in eutrophied water bodies worldwide.

**Electronic supplementary material:**

The online version of this article (doi:10.1186/s12898-017-0130-3) contains supplementary material, which is available to authorized users.

## Background

Cyanobacteria are a common component in the diets of herbivores in freshwater ecosystems. Cyanobacteria often occur in eutrophied water bodies and represent a low-quality food source for consumer species caused by a variety of factors such as the possession of toxins [1, 2], feeding inhibitors [3], unsuitable morphology [1] and the lack of essential dietary lipids [4]. In particular, their low amounts of the essential polyunsaturated fatty acids (PUFA) omega 3 and omega 6 [5] and sterols, strongly constrain the fitness of herbivores on cyanobacterial diets [4, 6]. Within a cyanobacterial species, individual strains can be toxic or non-toxic. Although less frequently studied, non-toxic cyanobacteria are known to reduce growth [7] and reproduction [8], at a similar magnitude as toxin-bearing cyanobacteria, to cladocerans and copepods. Surveys conducted in different parts of the world showed that up to 75% of cyanobacterial blooms can be non-toxic [9–11]. Non-toxic cyanobacteria may consequently impact ecosystems, trophic cascades and geochemical cycles [12]. Interestingly, in a meta-analysis by Wilson et al. [1], it was found that cyanobacterial toxins were actually less important with respect to their negative effects on consumer fitness than cyanobacterial cell morphology. Wilson et al. [1] therefore concluded that the role of cyanobacterial toxins in the determination of food quality may be less important than widely assumed and suggested that future research should focus more on nutritional deficiencies, morphology, and the toxicity of undescribed cyanobacterial compounds as mediators of the poor food quality of cyanobacteria.

Due to the multiple threats that cyanobacteria may pose to ecosystems, various attempts have been made to control cyanobacterial mass abundances (‘blooms’) through herbivory [13, 14]. However, this requires that the herbivores are able to efficiently utilize cyanobacteria as a food resource. While pure cyanobacteria may be a low quality food resource, a mixed diet with cyanobacterial and eukaryotic components may be easier to assimilate for many herbivores. For example, a severe sterol limitation of the planktonic herbivore *Daphnia* is assumed to occur only if cyanobacteria make up more than 80% of phytoplankton biomass [15]. Moreover, the growth of *Dreissena polymorpha* was strongly reduced while feeding upon a pure cyanobacterial diet deficient in polyunsaturated fatty acids (PUFAs) in comparison to a mixed diet rich in PUFAs [16]. By consuming a mixed diet, herbivores may obtain all nutrients required for growth. This is called the balanced diet hypothesis [17, 18] and has been described for numerous herbivores for example insects [19], snails [20] and fish [21]. Diet mixing may enable grazers to feed upon cyanobacteria without any significant decrease in fitness, and thus reduce cyanobacterial blooms. In a study by DeMott and Müller-Navarra [22], *Daphnia* feeding upon non-toxic cyanobacteria did not display any increase in growth, but when supplemented with a green alga a significant increase in growth was observed. Additionally, rotifers were found to grow better on a diet consisting of a mixture between green algae and cyanobacteria than on either single algal diet [23]. Herbivores may also compensate for a low quality diet through compensatory feeding [24–26]. This is a strategy in which herbivores increase their consumption rate as dietary nutrient concentrations decrease in order to maintain a sufficient uptake of the limiting nutrient(s). Compensatory feeding may thereby increase the fitness of herbivores, but it may also be associated with costs [27]. With respect to macronutrients, herbivores maintain a rather strict homeostasis [28, 29], for instance they need to maintain their body’s elemental composition by excreting excess nutrients. This requires energy and results in a reduction of fitness [30, 31]. Moreover, when consuming food in higher quantities, the dosage of potential toxins in the diet can increase [32].

Effects of cyanobacterial diets on freshwater gastropods have rarely been studied. While feeding upon benthic biofilms gastropods rarely only encounter cyanobacteria, but mixtures of various microalgae, bacteria and protozoa embedded in a mucopolysaccharide matrix [33]. Gastropods can represent up to 60% of the total biomass of macroinvertebrates in freshwater ecosystems [34] and they play a key role in the top-down control of benthic primary production. For instance, *Lymnaea stagnalis* (L.), is a benthic herbivore [34] and important grazer in freshwater habitats [35]. It is often found in small eutrophic water bodies [36] where cyanobacteria are extremely common. It is thus plausible to assume that *L. stagnalis* has evolved strategies to cope with cyanobacterial presence in its diet. *L. stagnalis* detects its food via semiochemicals [37], but prey selection on the level of individual food items (e.g. algal cells) is not possible due to the rather unspecific ingestion mode via the gastropod radula [35]. Moreover, it is a common model organism in experimental ecology [37–39].

The aim of this study was to test whether gastropods may be able to feed upon non-toxic cyanobacteria without any significant decrease in fitness through the benefits of diet-mixing. Further, we aimed to investigate which factors are responsible for the typically observed low food quality of non-toxic cyanobacteria to freshwater herbivores [40, 41]. We thus hypothesized (1) that a pure diet consisting of non-toxic cyanobacteria will decrease the fitness of freshwater gastropods, and that (2) diet-mixing allows these gastropods to feed upon non-toxic cyanobacteria without any significant decrease in fitness. To test our hypotheses, we conducted a laboratory experiment using juveniles of the great pond snail *L. stagnalis* which we fed with either single, non-toxic cyanobacterial and algal diets or a mixture of all (six) primary producer species. The aquatic primary producers chosen for this experiment belonged to the three most important groups of organisms in freshwater biofilms: cyanobacteria, chlorophytes and diatoms.

## Methods

### Cultivation

We randomly selected six species of primary producers, two chlorophytes (*Aphanochaete repens* and *Klebsormidium flaccidum*), two cyanobacteria (*Cylindrospermum* sp. *and Lyngbya halophila*), and two diatoms (*Navicula* sp. *and Nitzschia communis*) from the Culture Collection of Algae at Cologne (CCAC, see Table 1). The size and structure of the primary producer cells were determined by microscopy to test whether the morphology of the cells may impact the fitness of *L. stagnalis* (Table 1). All six primary producer strains were cultivated under continuous aeration in 8 L of cyanophyceae medium [42] for cyanobacteria and chlorophytes or in diatom medium [43] for diatoms, respectively. All cultures were kept in a climatized chamber at 20 ± 1 °C at a light intensity of 150 µmol photons s^−1^ m^−2^ as described elsewhere [44]. After one (chlorophytes and cyanobacteria) or two months (for diatoms) of exponential growth, the primary producers were harvested by centrifugation at 4500×*g* and the resulting pellets were freeze-dried [44]. Juvenile *L. stagnalis*, originating from a pond in Appeldorn, Germany, were raised in aquaria in a climatized chamber at 18 ± 1 °C with a light–dark period of 16:8 h and fed ad libitum with Tetra Wafer Mix™ fish food pellets (Tetra, Melle, Germany) prior to the experiment [44].

**Table 1:** The six benthic primary producers used in the experiment together with their cell shape, average biovolume and origin

Species	Origin/strain	Shape	Average biovolume (µm^3^)
*Aphanochaete repens*	CCAC/M2227	Sphere	350
*Klebsormidium flaccidum*	CCAC/2007 B	Cylinder	265
*Cylindrospermum* sp.	CCAC/1160 B	Cylinder	85
*Lyngbya halophila*	CCAC/1164 B	Cylinder, two half spheres	60
*Navicula* sp.	CCAC/1772 B	Prism on eliptic base	185
*Nitzschia communis*	CCAC/1762 B	Prism on eliptic base	410

The cell-specific biovolumes calculated on basis of the geometric shapes according to [45]. The shell morphology of the primary producer species were estimated as it may impact the ingestion by herbivores

### Growth experiment

A total of 64 juvenile *L. stagnalis* with a shell height (defined as the distance from the apex to the lower edge of the aperture) of 2.0 ± 0.2 mm were selected. Out of these, eight had their shells removed under a dissecting microscope and were subsequently freeze-dried for the determination of their initial soft body dry mass (dm) using a microbalance (Mettler UTM2, Giessen, Germany). The experiment took place in a climatized chamber at 20 ± 1 °C. The snails were individually kept in square polyethylene containers (length = 11 cm) with each 100 ml aged and aerated tap water and fed on a daily basis. The primary producers were rehydrated in 1 ml water each and then added through a hollow glass cylinder (d = 2.3 cm, h = 2.5 cm) that was submerged halfway in the water in the center of the snails’ container. After approximately 1 h when the primary producers had sedimented to the bottom of the container, the glass cylinders were removed. This yielded one clear resource patch and prevented selective feeding by the snails in the mixed primary producer treatment. Water was exchanged daily and the containers were replaced with clean ones every other day. The experiment consisted of seven treatments with eight replicates each, we thus used 56 containers, each containing one *L. stagnalis* individual. The seven treatments consisted of snails fed with a mixture of all six primary producer species or with one of the six primary producer species. To ensure that the snails could feed ad libitum, the amount of added food was increased during the 33 days experimental duration using previously estimated shell size specific ingestion rates [44]. The shell height of the snails was measured every three days as described above. After 33 days, the soft body dry mass of the remaining 54 snails (single incidents of death had occurred in the treatments with *A. repens* and *K. flaccidum* as diet organism during the experiment) was determined as described above. Since the juvenile growth of *L. stagnalis* can be assumed to be exponential [44], the somatic growth rate of the snails was estimated using the following equation:

$$ g = \frac{{\ln (m_{end} ) - \ln \left( {m_{start} } \right)}}{time \left[ d \right]} $$

where m_start_ is the initial dry mass of snails and m_end_ is the dry mass of the snails at the end of the experiment (day 33) over time (d), yielding the somatic growth rate (g).

### Ingestion rate

On the final day of the experiment, the snails’ ingestion rates were determined in the same setup used to determine the somatic growth rates of the snails. Additionally, three control units per dietary treatment were set up without snails. After 19 h, the snails and their fecal pellets were separately removed from the containers and the remaining primary producers were filtered onto pre-combusted glass fiber filters (GF/F, d = 25 mm, VWR GmbH, Darmstadt, Germany) and dried at 60 °C for 24 h. The amount of ingested food was determined by subtracting the primary producer dry mass remaining in the snails’ containers after 19 h from the primary producer dry mass in the consumer-free controls.

### Elemental analysis

To determine the C:N ratios of the primary producers, approximately 1 mg of each freeze-dried primary producer culture, including a mixture of all six primary producers in equal dry mass were packed into tin capsules (HekaTech, Wegberg, Germany) and subsequently analyzed using a Thermo Flash EA 2000 elemental Analyser (Schwerte, Germany). For the analysis of particulate phosphorus, approximately 1 mg of freeze-dried primary producers were transferred into a solution of potassium peroxodisulphate and 1.5% sodium hydroxide. The solution was subsequently autoclaved for 1 h at 120 °C and the soluble reactive phosphorus was analyzed using the molybdate–ascorbic acid method [46]. Both analyses were replicated fivefold per food treatment. The same method was applied to separately determine the molar C:N:P ratios of the 54 snails. When the mass of individual gastropod samples did not reach the required 1 mg dry mass, samples of several individuals from the same treatment were pooled randomly to reach sufficient sample masses.

### Fatty acid analysis

Cultures of all primary producers were harvested in the exponential growth phase and filtered in triplicate onto precombusted GF/F filters to assess their fatty acid contents. Filters were placed into 5 mL CH_2_Cl_2_/MeOH (2:1 v/v) to extract total lipids. The samples were incubated over night at 4 °C whereafter 10 µg of methyl heptadecanoate (C17:0 ME) and 5 µg methyl tricosanoate (C23:0 ME), were added as internal standards. Subsequently, the samples were homogenized in an ultrasonic bath for 1 min and then centrifuged at 4500×*g* for 5 min. Afterwards, the supernatants were dried at 40 °C under a gentle stream of nitrogen. Hydrolysis of lipids and subsequent methylation of fatty acids were achieved by adding 5 mL of 3 N methanolic HCl (Supelco) to the sample and then incubating the sample for 20 min at 70 °C to yield fatty acid methyl esters (FAMEs). The FAMEs were extracted using 6 mL isohexane. The hexane phase was again dried at 40 °C under a gentle stream of nitrogen. Finally, all samples were dissolved in 100 µL isohexane. The samples were then subjected to gas chromatographic analyses on a 6890 N GC System (Agilent Technologies, Waldbronn, Germany) equipped with a DB-225 capillary column (30 m, 0.25 mm i.d., 0.25 µm film thickness, J&W Scientific, Folsom, CA, USA) and a flame ionization detector (FID). The conditions of the GC were as follows: injector and FID temperatures were set to 220 °C; the initial oven temperature were 60 °C for 1 min, followed by a 2 min temperature ramp to 180 °C, then the temperature was increased to 200 °C over a time period of 12.9 min followed by a final 20.6 min temperature increase to 220 °C; helium (5.0 purity) with a flow rate of 1.5 ml min was used as carrier gas. FAMEs were identified by comparison of retention times with those of reference compounds and quantified using the internal standard and previously established calibration functions for each individual FAME. For more details we refer the reader to [47].

### Toxin analysis

The two cyanobacterial species used in the experiment (*L. halophila* and *Cylindrospermum* sp.) are known to sometimes contain the toxins cylindrospermopsin and lyngbyatoxin-a. We therefore used high-resolution LC–MS to screen for the cyanobacterial toxins cylindrospermopsin and lyngbyatoxin-a in the cyanobacterial cultures to ensure that the toxins were not produced by the cyanobacterial strains. To screen the two cyanobacteria for the toxins cylindrospermopsin and lyngbyatoxin-a, a crude extract from the freeze-dried samples (500 mg dry mass) of the two cyanobacteria (*Cylindrospermum* sp. and *L. halophila*) were prepared using 50 mL of 100% methanol (HPLC Grade, VWR). The extracts were incubated for 1 h on a rotary shaker and subsequently centrifuged at 4500×*g* for 5 min. The supernatant was then evaporated and the residue dissolved in 5 mL of 100% methanol. Three 5 µL subsamples each were analyzed on an Accela Ultra high pressure liquid chromatography (UPLC) system (Thermo Fisher) coupled with an exactive orbitrap mass spectrometer (Thermo Fisher) with electrospray ionization (ESI) in positive and negative ionization mode. As stationary phase, a Nucleosil C18 column (2 × 125 mm length, pore size 100 Å, particle size 3 μm; Macherey–Nagel, Düren, Germany) was used with a gradient of acetonitrile (ACN) and ultrapure water, each containing 0.05% trifluoroacetic acid (TFA). The column temperature was set to 30 °C and the flow rate to 300 µL/min. The solvent gradient started with 0 min: 38% ACN; 2 min: 40% ACN; 12 min: 50% ACN; 12.5 min: 100% ACN; 15 min: 100% ACN; 15.5 min: 38% ACN; 17 min: 38% ACN. Mass spectrometry was carried out at 1 scan s^−1^ from 150 to 1500 Da in positive and from 120 to 1500 Da in negative ionization mode, respectively (spray voltage 4.5 kV pos./4.3 kV neg. capillary temperature 325 °C, sheath and aux gas nitrogen set to 40 pos./35 neg. and 15 pos./5 neg. respectively). For the identification of potential cyanobacterial toxins, we used extracted ion chromatograms for the respective specific masses of the different compounds (Cylindrospermopsin pos. 416.12345 Da/neg. 414.10889 Da, Lyngbyatoxin-a pos. 438.31150 Da/neg. 436.29695 Da) granting a maximum mass deviation of 3 parts per million (ppm). Electrospray ionization resulted in adduct ions with one positive/one negative charge for the two compounds. We used the Xcalibur software package (Thermo Fisher) for qualitative analysis.

### Statistics

The gastropods’ shell height increase over time was analysed via repeated measures ANOVA in R (v. 3.3.1) [48], followed by Bonferroni correction. All other statistical tests were performed in SigmaPlot (v. 11, SysStat). The data were checked for normal distribution using the Shapiro–Wilk’s test and for homoscedasticity using Levene’s test. When the data fulfilled the criteria for a parametric test, one-way ANOVAs was performed followed by Tukey’s HSD. When the data did not fulfil the criteria for an ANOVA, a Kruskal–Wallis test was performed followed by Dunn’s post hoc test. Linear regressions were performed to test for relationships between the algal C:N ratio and the ingestion rate of the snails, the algal C:N ratio and the somatic growth rates of the snails, the molar C:N, C:P, N:P ratios of the algae and the molar C:N, C:P, N:P ratios of the snails, and the biovolume of the algae and the somatic growth rate of the snails. An exponential growth, single, 2 parameter regression was performed to test for the relationship between snail dry mass and shell height.

## Results

**Fig. 1:**
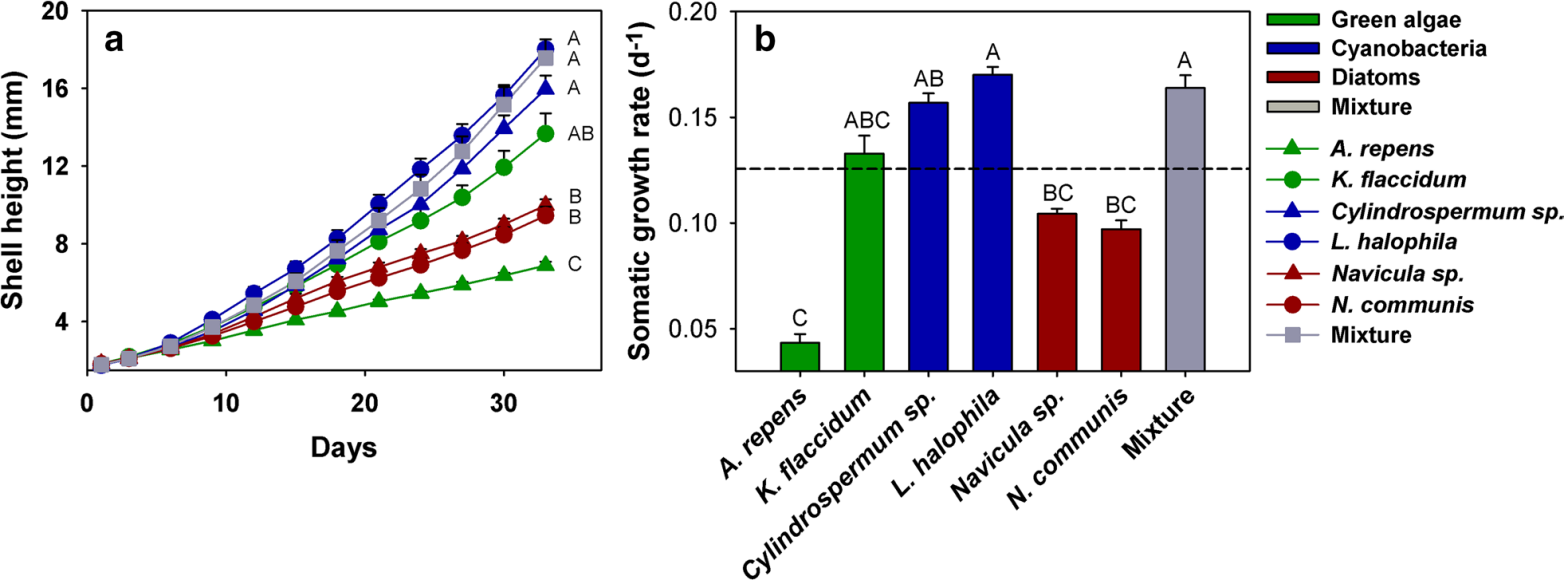
Increase of shell height (**a**) over time and somatic growth rate (**b**) of *L. stagnalis.* The snails were fed a diet consisting of single primary producer species or a mixture of all six species (Mixture) in equal biomass (mean + 1 SE; N = 7–8). The *dashed line* indicates the average of all single algal treatments; means which were found to be significantly different in Tukey post hoc comparisons are labelled with *different letters*

*Lymnaea stagnalis* fed the cyanobacteria or a mixed diet grew faster than on any of the pure eukaryotic algae, except for the comparison with *K. flaccidum* (repeated measures ANOVA, F = 26.6, df = 6, P < 0.001, Fig. 1a). The somatic growth rate of *L. stagnalis* was significantly higher in the treatments where *L. halophila* and the mixed treatment was offered compared to the treatments in which *L. stagnalis* was provided with diatoms (Fig. 1b).

**Fig. 2:**
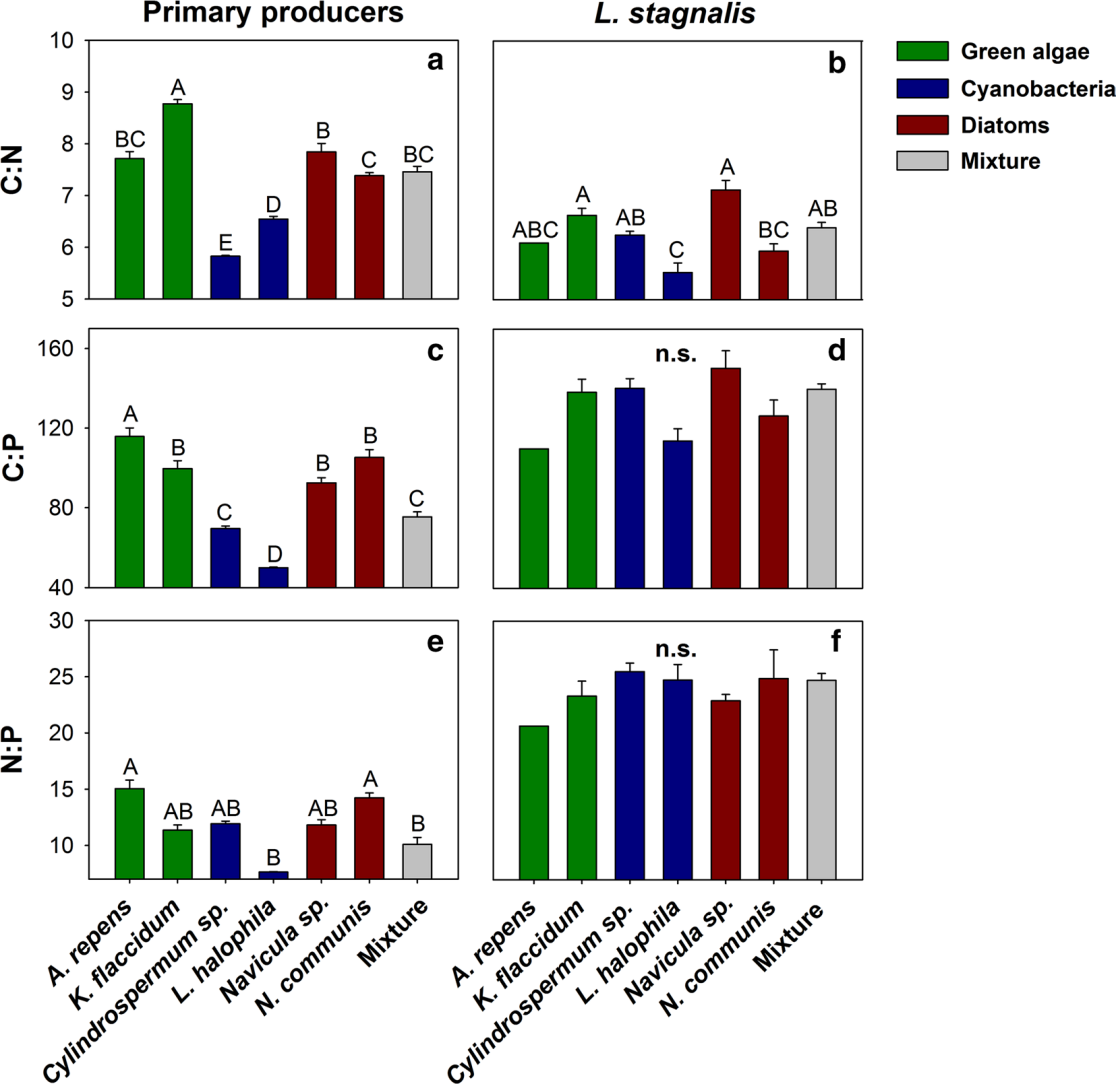
Molar C:N:P ratios of the primary producers and of *L. stagnalis*. C:N:P ratios of single primary producer species or of the mixed diet (**a**,** c**,** e**, mean + 1 SE; N = 5) and of *L. stagnalis* feeding upon the single and mixed diets (**b**,** d**,** f**, mean + 1 SE; N = 1–6). Means which were found to be significantly different in Tukey post hoc comparisons are labelled with *different letters*

The molar C:N ratio (one-way ANOVA, F = 100.00, df = 6, P < 0.001, Fig. 2a) and the molar C:P ratio (one-way ANOVA, F = 59.16, df = 6, P < 0.001, Fig. 2c) of the cyanobacteria were significantly lower than that of the algae. The N:P ratio of *L. halophila* were significantly lower than the N:P ratio of *A. repens* and *N. communis* (Kruskal–Wallis, H = 31.24, df = 6, P < 0.001, Fig. 2e). The C:N ratios of the snails varied significantly between the diet treatments (one-way ANOVA, F = 14.01, df = 5, P < 0.001, Fig. 2b). *L. stagnalis* feeding upon *L. halophila* had a significantly lower C:N ratio compared to all other treatments (Fig. 2b), except for *A. repens* and *N. communis*. While C:N ratios were lower in *L. stagnalis* compared to their diets (Fig. 2a, b), the C:N:P ratios of the snails and their dietary organisms were not significantly correlated (see Additional file 1).

**Fig. 3:**
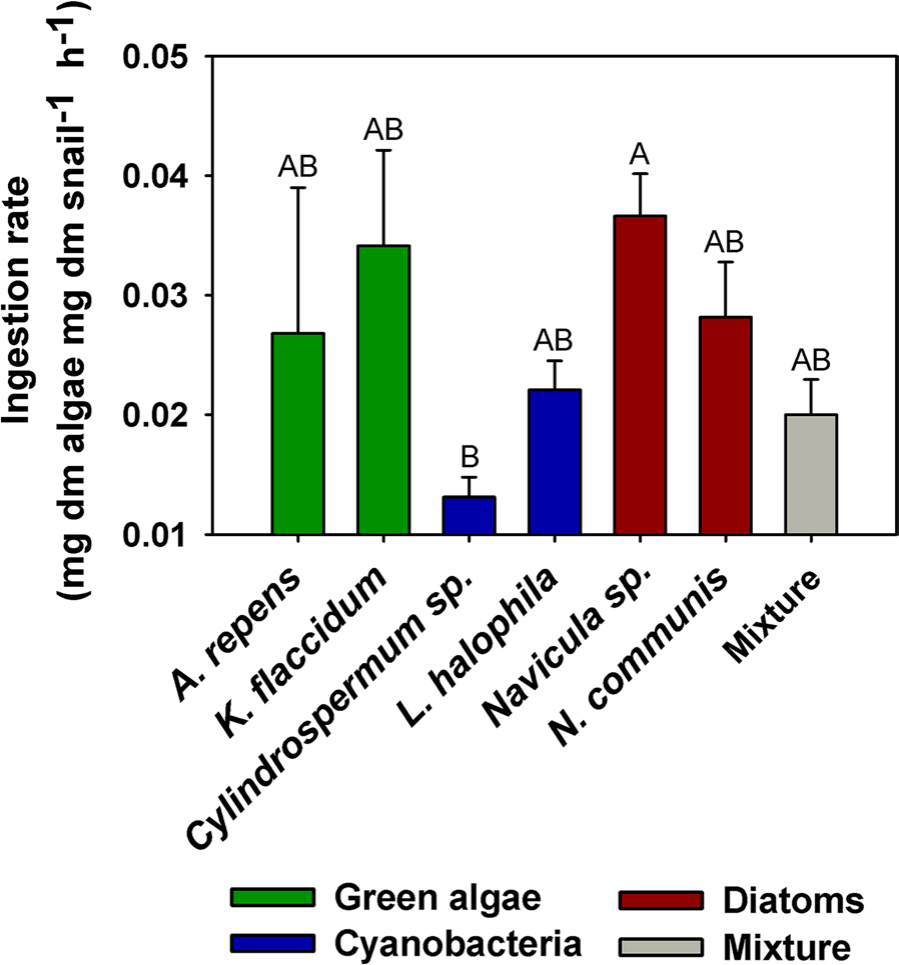
Mass-specific ingestion rates of *L. stagnalis*. Ingestion rates (mean + 1 SE; N = 7–8) were determined on the last day of the growth experiment. Means which were found to be significantly different in Tukey post hoc comparisons are labelled with *different letters*

**Fig. 4:**
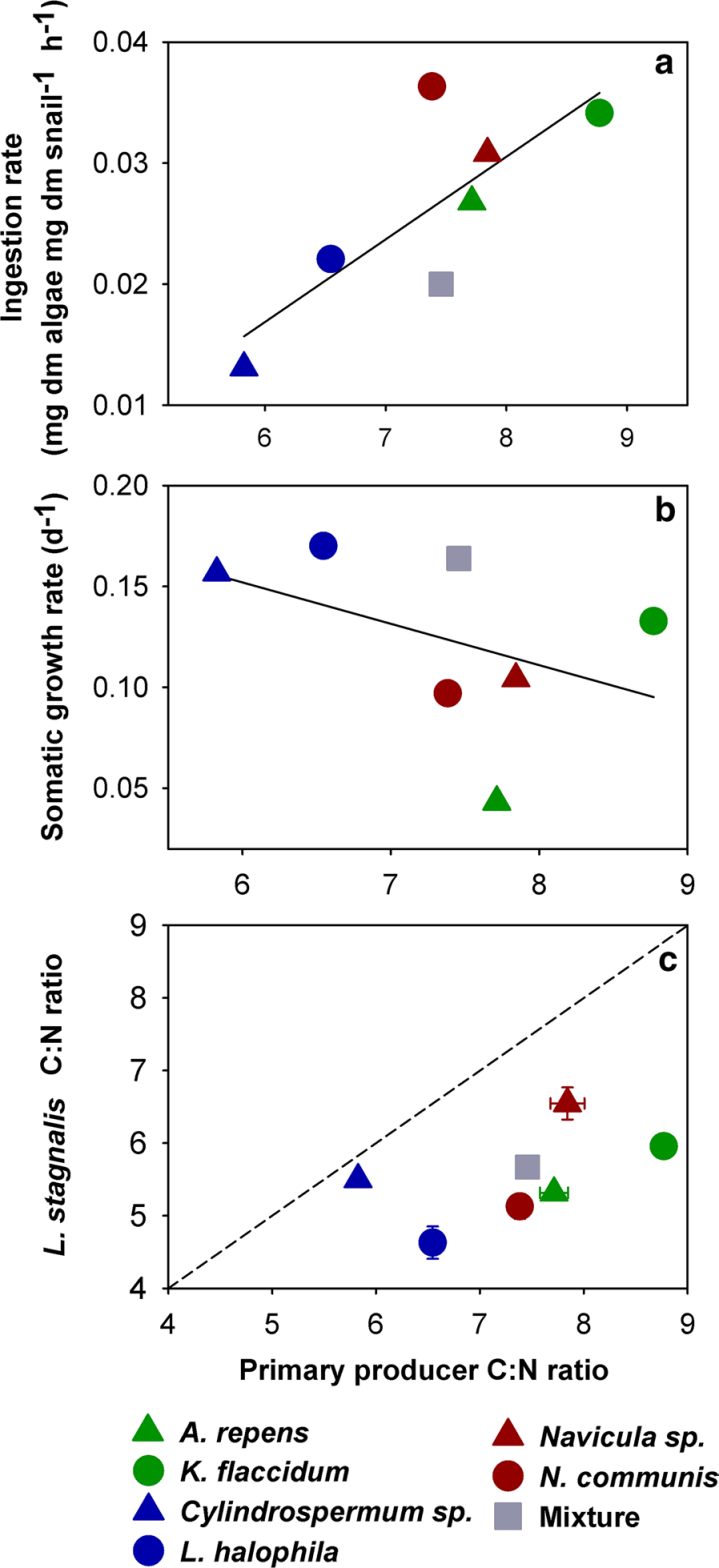
Relationships of primary producer C:N ratio with various parameters of *L. stagnalis*. The average C:N ratio of the algae (*red* for diatoms and *green* for chlorophytes), cyanobacteria (*blue*) and the mixed diet (*grey*) in each treatment (**a**, **b**, N = 7) is plotted versus the average ingestion rate of *L. stagnalis* in the respective treatment (**a**, N = 7), the average snail somatic growth rate in the respective treatment (**b**, N = 7), or the C:N ratio of the algae and cyanobacteria in each treatment (**c**, mean ± 1 SE; N = 7) versus ingestion rate of *L. stagnalis* in the respective treatment (**c**, mean ± 1 SE; N = 7)

The food consumption of *L. stagnalis* was significantly higher when offered *Navicula* sp. compared to the treatment in which the snails fed on *Cylindrospermum* sp. (Kruskal–Wallis, H = 19.88, df = 6, P = 0.003, Fig. 3). However, no significant differences of the ingestion rates between any other treatments were found (Fig. 3). Moreover, the mean ingestion rate of the snails in each treatment increased linearly with the dietary C:N ratio (y = −0.0241 + (0.00683×), R^2^ = 0.61, df = 6, P = 0.039, N = 7; Fig. 4a), but the mean somatic growth rate and the dietary C:N ratio did not correlate (linear regression, x = 8.458 − (8.825y), R^2^ = 0.18, df = 6, P = 0.34, N = 7; Fig. 4b). The mean C:N ratios of the snails in each treatment were lower than the mean C:N ratios of any of the diets (N = 7; Fig. 4c). Additionally, no significant relationships between somatic growth rate and the dietary organisms’ cell sizes were found (see Additional file 2).

The fatty acid concentration of the primary producers differed, *A. repens* and *Cylindrospermum* sp. were particularly rich in α-linolenic acid (C 18:3 n − 3) whereas *N. communis* was rich in eicosapentaenoic acid (C20:5 n − 3, see Additional file 3). *A. repens* and the cyanobacteria contained the highest absolute amount of palmitic acid (C 16:0, see Additional file 3), while the *A. repens* and *N. communis* contained the highest total amounts of fatty acids and PUFAs (see Additional file 4).

We did not detect the cyanobacterial toxin cylindrospermopsin in the cyanobacterium *Cylindrospermum* sp. nor lyngbyatoxin-a in *L. halophila* via high-resolution LC–MS.

## Discussion

Contrary to our hypothesis, a mixed diet containing non-toxic cyanobacteria did not provide a higher growth rate for *L. stagnalis* compared to pure non-toxic cyanobacterial diets. Surprisingly, the single non-toxic cyanobacteria provided growth rates higher than or equal to the diet consisting of mixed pro- and eukaryotic primary producers. Non-toxic cyanobacteria—at least the two strains investigated here—may therefore be considered a high quality resource for *L. stagnalis*. Negative effects of non-toxic cyanobacteria on the fitness of animals have frequently been reported [7, 8]. However, a screening of cyanobacterial strains demonstrated that some cyanobacterial strains can have a high nutritional value [49]. The importance of the supply ratio or stoichiometry of carbon (C), nitrogen (N) and phosphorus (P) is well studied, as the balance or imbalance in the molar C:N:P supply ratio has been linked to herbivore growth [50, 51], fecundity [52, 53], developmental times [52] and survival rates [52]. While nitrogen is mainly needed for protein synthesis [29], phosphorus is required for the synthesis of phospholipids and nucleic acids [54]. We found that the cyanobacterial species exhibited slightly lower C:N and C:P ratios than the green algae. On the other hand, the overall differences in nutrient stoichiometry between the diet organisms were not particularly pronounced and the nutrient ratios typically below those observed for *L. stagnalis*, which makes a direct nitrogen or phosphorus limitation of snail growth on the green algal diets unlikely. Furthermore, we did not find a significant correlation between the C:N ratio of the algae and the somatic growth rate of the snails, suggesting that the snails were not nitrogen limited. Also, the C:P ratios and the C:N ratios of the snails and the primary producers did not correlate.

The ingestion rate of the snails increased linearly with the C:N ratio of the primary producers, suggesting that compensatory feeding occurred. However, the somatic growth rate of the snails did not correlate with the C:N ratio of the primary producers. It is possible that compensatory feeding by *L. stagnalis* decreased a reduction in growth caused by nitrogen limitation in our experiment. In a previous study, it was found that the freshwater snail *Radix ovata* displayed compensatory feeding on low nutrient diets [24]. This dampened the differences in fitness compared to the treatments in which *R. ovata* was fed a nutrient-rich diet.

The lack of essential fatty acids and sterols in cyanobacteria is frequently held responsible for the reduction in growth of pelagic herbivorous zooplankton [4, 15], but also of filter-feeding clams *Corbicula* [40] and mussels *Dreissena* [55]. Even though the diatom diets in our experiment contained much more PUFAs than the cyanobacteria, *L. stagnalis* grew better on both cyanobacteria compared to any of the diatoms. This supports previous findings that lymnaeid gastropods appear to be less susceptible to dietary PUFA limitations than other freshwater invertebrates [56].

Difference in morphology [1, 57, 58] and cell size [44] can strongly influence the ingestion of algae and cyanobacteria by herbivores. We found that snails feeding upon two filamentous cyanobacterial species grew best. Similar results have been found for the lymnaeid species *Radix peregra* which ingests filamentous green algae better than diatoms [58]. Grazing by *Lymnaea elodes* for instance increased the abundance of small coccoid cells of green algae and cyanobacteria at the expense of larger diatoms [59], suggesting a preference for larger algal cells. However, we could not find any clear correlation between algal cell size and the somatic growth rate of *L. stagnalis* in this study.

If the fitness of *L. stagnalis* is increased by the consumption of non-toxic cyanobacteria rather than green algae and diatoms, *L. stagnalis* might have the ability to decrease non-toxic cyanobacterial abundances. In fact, it has been found that snails have the potential to reduce cyanobacterial blooms. In a study by Armitage and Fong [60], primary producers were subjected to nutrient enrichment which led to an increase in cyanobacterial blooms by up to 200%. When snails were allowed to feed upon the primary producers only cyanobacteria decreased in biomass [60]. The snails did not avoid consumption of the toxic cyanobacteria, which indicated that they perceived the cyanobacteria as a suitable food resource, even though cyanobacteria could be linked to an increased mortality of the snails [60]. Toxic cyanobacteria are likely to influence competitive interactions by consumer species favoring the most tolerant ones [61]. Previous studies found that crustacean zooplankton of the genus *Daphnia* are able to locally adapt to environments where cyanobacteria occur in high abundances [62–64]. Similarly physical adaptations in freshwater mussels to cyanobacterial toxins have been found [65]. As *L. stagnalis* often occur in eutrophicated water bodies [36], habitats where cyanobacteria are known to occur, it is possible that—similar to *zooplankton and mussels*—*Lymnaea* have evolved to coexist with cyanobacteria.

We feed *L. stagnalis* with single primary producer species or a mixture of all species. Due to time constraints we did not investigate the effects of all diet combinations possible on the fitness of *L. stagnalis*, however, the experimental set-up still it provided insights into gastropod-cyanobacteria relationships.

## Conclusions

Efforts have been made in order to control cyanobacterial abundances by manipulating the biomass of herbivores and thereby increasing the top–down grazing pressure on cyanobacteria. However, most studies conducted used toxic cyanobacterial species; therefore, the knowledge of non-toxic cyanobacteria-grazer relationships remains limited. We hypothesized that the growth rate of *L. stagnalis* should be significantly reduced when feeding upon non-toxic cyanobacteria, we found however, the opposite pattern. Non-toxic cyanobacteria and the mixed diet provided the best growth rates for the snails. *L. stagnalis* might thus be a good biological control agent for non-toxic cyanobacterial mass occurrences. These results hence have considerable repercussions to how the dietary quality of non-toxic cyanobacteria for gastropods is perceived.

## Additional files

**Additional file 1.** Relationship between the C:N:P ratios (mean ± SE) of the primary producers and *L. stagnalis*. Nonsignificant linear regressions for C:N (A, y = 3.006 + (0.335 x), R^2^ = 0.27, df = 6, P = 0.23), C:P (B, y = 134.678 - (0.0388 x), R^2^ < 0.005, df = 6, P = 0.90), and N:P (C, y = 27.849 - (0.342 x), R^2^ = 0.26, df = 6, P = 0.25).

**Additional file 2.** Relationship between primary producer biovolume and somatic growth rate of *L. stagnalis*. Not statistically significant linear regression, y = 0.176 - (0.0000260 x), R^2^ = 0.63, df = 5, P = 0.06.

**Additional file 3.** A table of the fatty acid composition of the primary producers used as a food resource for *L. stagnalis*. Values given are means ± 1 SE of N = 3 replicates analyzed via gas chromatography of fatty acid methyl esters (n.d. = not detected), the standard errors are given in parentheses.

**Additional file 4.** Total fatty acid and polyunsaturated fatty acid (PUFA) concentration of the primary producers. Values given are means ± 1 SE of N = 3 replicates analyzed via gas chromatography of fatty acid methyl esters, the standard errors are given in parentheses.
