# Supplementary figures and images for: High dietary quality of non-toxic cyanobacteria for a benthic grazer and its implications for the control of cyanobacterial biofilms
Source: BMC Ecol. 2017 May 18;17:20. doi: 10.1186/s12898-017-0130-3 (PMC5437396; doi:10.1186/s12898-017-0130-3)

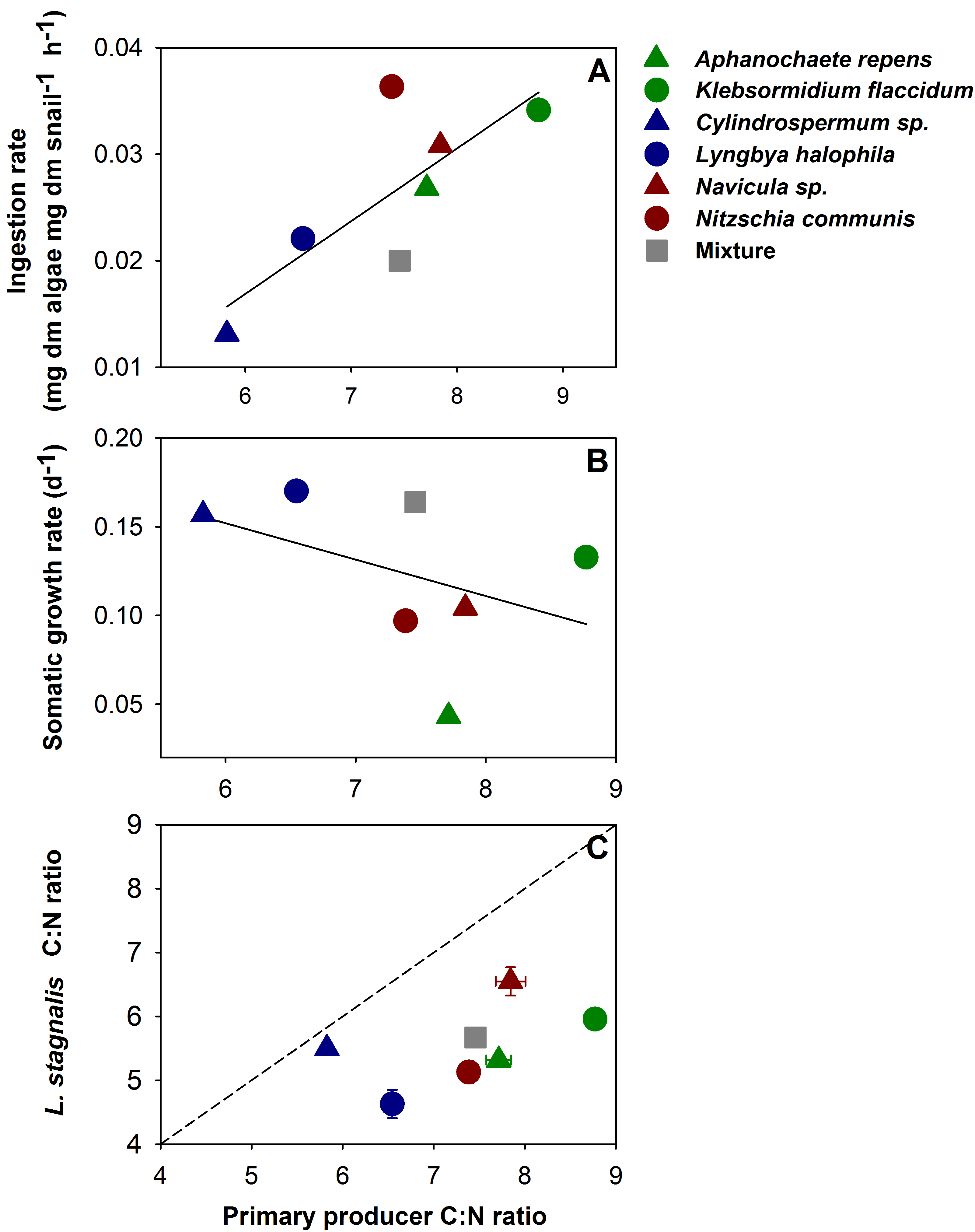

Supplement: Supplementary file 1 — Additional file 1. Relationship between the C:N:P ratios (mean ± SE) of the primary producers and L. stagnalis. Nonsignificant linear regressions for C:N (A, y = 3.006 + (0.335 x), R2 = 0.27, df = 6, P = 0.23), C:P (B, y = 134.678 - (0.0388 x), R2 < 0.005, df = 6, P = 0.90), and N:P (C, y = 27.849 - (0.342 x), R2 = 0.26, df = 6, P = 0.25). [file 12898_2017_130_MOESM1_ESM.tiff]

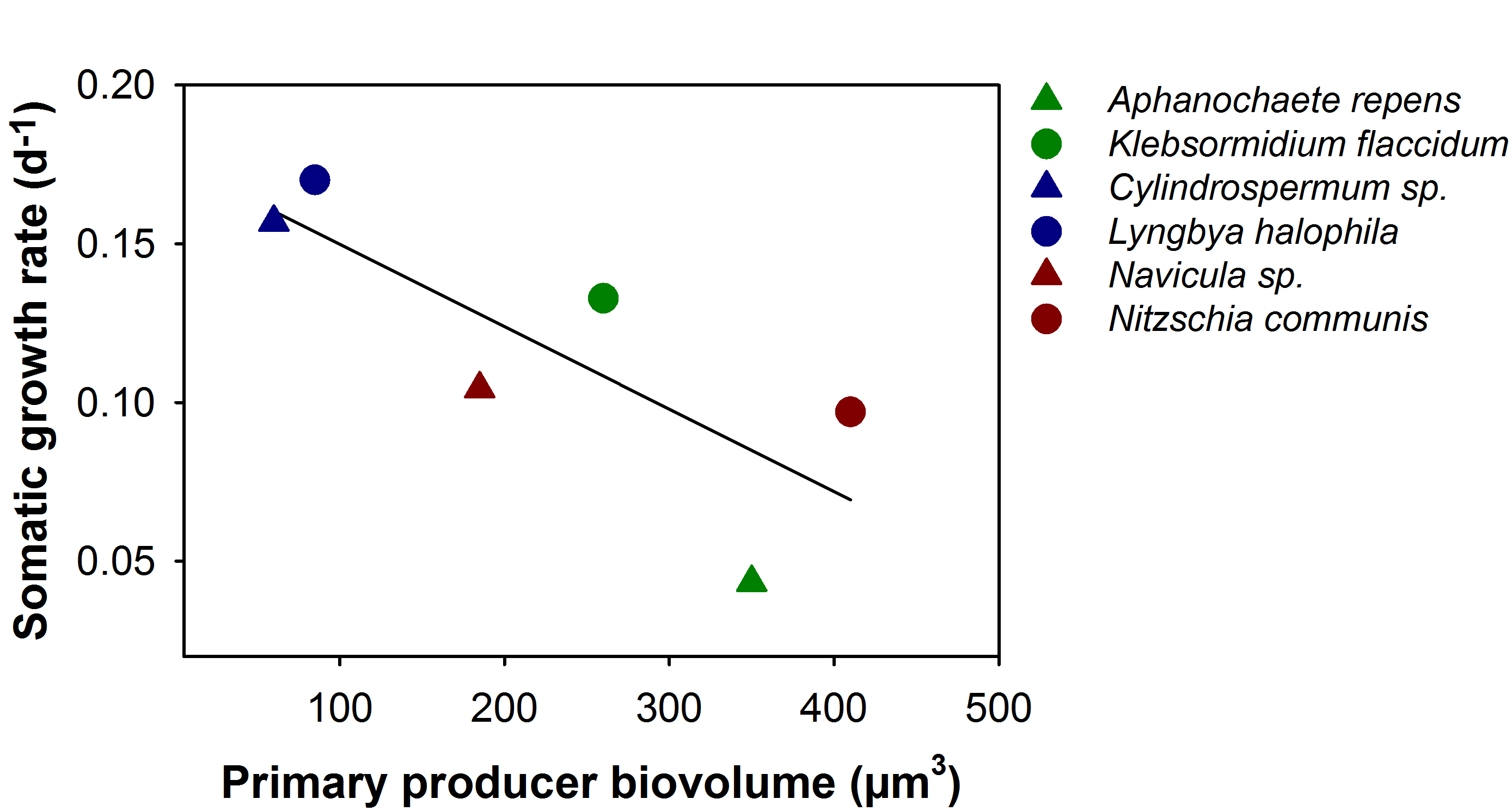

Supplement: Supplementary file 2 — Additional file 2. Relationship between primary producer biovolume and somatic growth rate of L. stagnalis. Not statistically significant linear regression, y = 0.176 - (0.0000260 x), R2 = 0.63, df = 5, P = 0.06. [file 12898_2017_130_MOESM2_ESM.tiff]
