# Supplementary material for: High dietary quality of non-toxic cyanobacteria for a benthic grazer and its implications for the control of cyanobacterial biofilms
Source: BMC Ecol. 2017 May 18;17:20. doi: 10.1186/s12898-017-0130-3 (PMC5437396; doi:10.1186/s12898-017-0130-3)
Supplement: Supplementary file 3 — Additional file 3. A table of the fatty acid composition of the primary producers used as a food resource for L. stagnalis. Values given are means ± 1 SE of N = 3 replicates analyzed via gas chromatography of fatty acid methyl esters (n.d. = not detected), the standard errors are given in parentheses. [file 12898_2017_130_MOESM3_ESM.docx]

|  | **Fatty acid content (µg mg C^-1^)** | | | | | |
| --- | --- | --- | --- | --- | --- | --- |
| **Fatty acid** | ***Aphanochaete repens*** | ***Klebsormidium flaccidum*** | ***Cylindro- spermum sp.*** | ***Lyngbya  halophila*** | ***Navicula sp.*** | ***Nitzschia  communis*** |
| **C 16:0** | 38.42 (1.78) | 4.95 (0.21) | 29.54 (0.90) | 41.44 (1.46) | 12.93 (1.17) | 22.75 (1.77) |
| **C 16:1 ω9** | 2.27 (0.02) | 0.78 (0.04) | 3.65 (0.16) | 2.87 (0.05) | 9.47 (0.04) | 76.62 (6.28) |
| **C 17:1 ω9** | 7.67 (0.16) | 0.10 (0.02) | n.d. | 1.39 (0.15) | 0.13 (0.13) | 0.31 (0.15) |
| **C 18:0** | 0.93 (0.13) | 0.52 (0.03) | 1.40 (0.05) | 7.50 (0.26) | 3.78 (0.39) | 1.37 (0.08) |
| **C 18:1 ω9 trans** | 2.34 (2.34) | 0.67 (0.03) | 1.14 (0.07) | 1.58 (0.02) | 5.56 (0.21) | 0.98 (0.04) |
| **C 18:1 ω9 cis** | 4.97 (0.40) | 0.17 (0.00) | 1.77 (0.32) | 1.97 (0.12) | 15.28 (0.57) | 1.39 (0.20) |
| **C 18:1 ω7** | 1.99 (1.99) | n.d. | 1.69 (0.85) | 5.05 (0.03) | n.d. | n.d. |
| **C 18:2 ω6 cis** | 33.56 (0.76) | 7.48 (0.23) | 11.65 (0.33) | 1.83 (0.07) | n.d. | 0.50 (0.13) |
| **C 18:3 ω6** | 4.16 (0.01) | 0.15 (0.01) | n.d. | 0.28 (0.02) | n.d. | 0.23 (0.03) |
| **C 18:3 ω3** | 47.63 (0.13) | 3.48 (0.10) | 31.66 (0.82) | 0.31 (0.04) | 0.30 (0.12) | 1.50 (0.14) |
| **C 18:4 ω3** | 9.13 (0.22) | n.d. | 0.11 (0.11) | 1.93 (0.01) | n.d. | 0.94 (0.06) |
| **C 20:4 ω6** | n.d. | 0.10 (0.01) | n.d. | n.d. | n.d. | 5.23 (0.69) |
| **C 20:5 ω3** | n.d. | 0.16 (0.10) | n.d. | n.d. | 1.01 (0.71) | 21.61 (2.36) |
| **C 22:0** | 2.23 (0.80) | 0.08 (0.02) | n.d. | n.d. | n.d. | n.d. |
| **C 22:6 ω3** | n.d. | n.d. | n.d. | n.d. | n.d. | 7.83 (0.73) |
| **C 24:0** | n.d. | n.d. | n.d. | n.d. | n.d. | 2.79 (0.28) |
