# Supplementary material for: High dietary quality of non-toxic cyanobacteria for a benthic grazer and its implications for the control of cyanobacterial biofilms
Source: BMC Ecol. 2017 May 18;17:20. doi: 10.1186/s12898-017-0130-3 (PMC5437396; doi:10.1186/s12898-017-0130-3)
Supplement: Supplementary file 4 — Additional file 4. Total fatty acid and polyunsaturated fatty acid (PUFA) concentration of the primary producers. Values given are means ± 1 SE of N = 3 replicates analyzed via gas chromatography of fatty acid methyl esters, the standard errors are given in parentheses. [file 12898_2017_130_MOESM4_ESM.docx]

| **Species** | **Fatty acid concentration**  **(µg mg C^-1^)** | **PUFA concentration**  **(µg mg C^-1^)** |
| --- | --- | --- |
| *Aphanochaete repens* | 159.56 (1.21) | 113.17 (2.11) |
| *Klebsormidium flaccidum* | 20.58 (0.25) | 13.01 (0.19) |
| *Cylindrospermum sp.* | 86.82 (2.15) | 50.59 (1.60) |
| *Lyngbya halophila* | 88.06 (2.75) | 13.2 (0.32) |
| *Navicula sp.* | 61.01 (2.35) | 32.37 (0.39) |
| *Nitzschia communis* | 152.19 (13.33) | 90.54 (7.32) |
